# Data on Fe (II) biosorption onto *Sargassum hystrix* algae obtained from the Persian Gulf in Bushehr Port, Iran

**DOI:** 10.1016/j.dib.2016.10.018

**Published:** 2016-10-29

**Authors:** Fatemeh Faraji Ghasemi, Sina Dobaradaran, Alireza Raeisi, Abdolhamid Esmaili, Mohammad Javad Mohammadi, Mozhgan Keshtkar, Sara Ghaderi Nasab, Farshid Soleimani

**Affiliations:** a Department of Environmental Health Engineering, Faculty of Health, Bushehr University of Medical Sciences, Bushehr, Iran; b The Persian Gulf Marine Biotechnology Research Center, The Persian Gulf Biomedical Sciences Research Institute, Bushehr University of Medical Sciences, Bushehr, Iran; c Systems Environmental Health, Oil, Gas and Energy Research Center, The Persian Gulf Biomedical Sciences Research Institute, Bushehr University of Medical Sciences, Bushehr, Iran; d The Persian Gulf Tropical Medicine Research Center, The Persian Gulf Biomedical Sciences Research Institute, Bushehr University of Medical Sciences, Bushehr, Iran; e Department of Environmental Health Engineering, School of Public Health and Environmental Technologies Research Center, Ahvaz Jundishapur University of Medical Sciences, Iran

**Keywords:** Biosorption, Bushehr, Iron, Persian Gulf, *Sargassum hystrix*

## Abstract

In this article, we used *Sargassum hystrix* algae as biosorbent for removal of Fe (II) from aqueous solutions that was collected along the Persian Gulf coastline, Bushehr, Iran. The concentration level of remaining Fe (II) in the samples was measured by using flame atomic absorption spectrometry (FAAS, Varian AA240, Australia). The isotherms, kinetics and modeling data of Fe (II) biosorption onto *Sargassum hystrix* were also presented.

**Specifications Table**

**Table:** 

*Subject area*	*Chemistry*
*More specific subject area*	*Biosorption*
*Type of data*	*Table, figure*
*How data was acquired*	*Flame Atomic Absorption Spectrometry (FAAS, Varian AA240, Australia)*
*Data format*	*Raw, analyzed*
*Experimental factors*	– After collection of algae along the Persian Gulf, it was washed 3 times by urban water and 2 times by deionized water to eliminate dirt and contaminants, then dried, powdered and sieved by using a screen. – The effects of contact times, initial concentrations of Fe (II) and different dosage of biosorbent were examined.
*Experimental features*	*Sargassum hystrix biomass as low cost biosorbent for removal Fe (II) ions*
*Data source location*	*Bushehr, Iran*
*Data accessibility*	*Data is with this article.*

**Value of the data**

• The data of *Sargassum hystrix* algae for Fe (II) removal from aqueous solution was described.
• Data show that brown algae can be used as low cost biosorbent for removal of other metals from aqueous solution.
• Data of this study can be used to design the bisorption experiments for removal of heavy metals.

## 1 Data

**Fig. 1:**
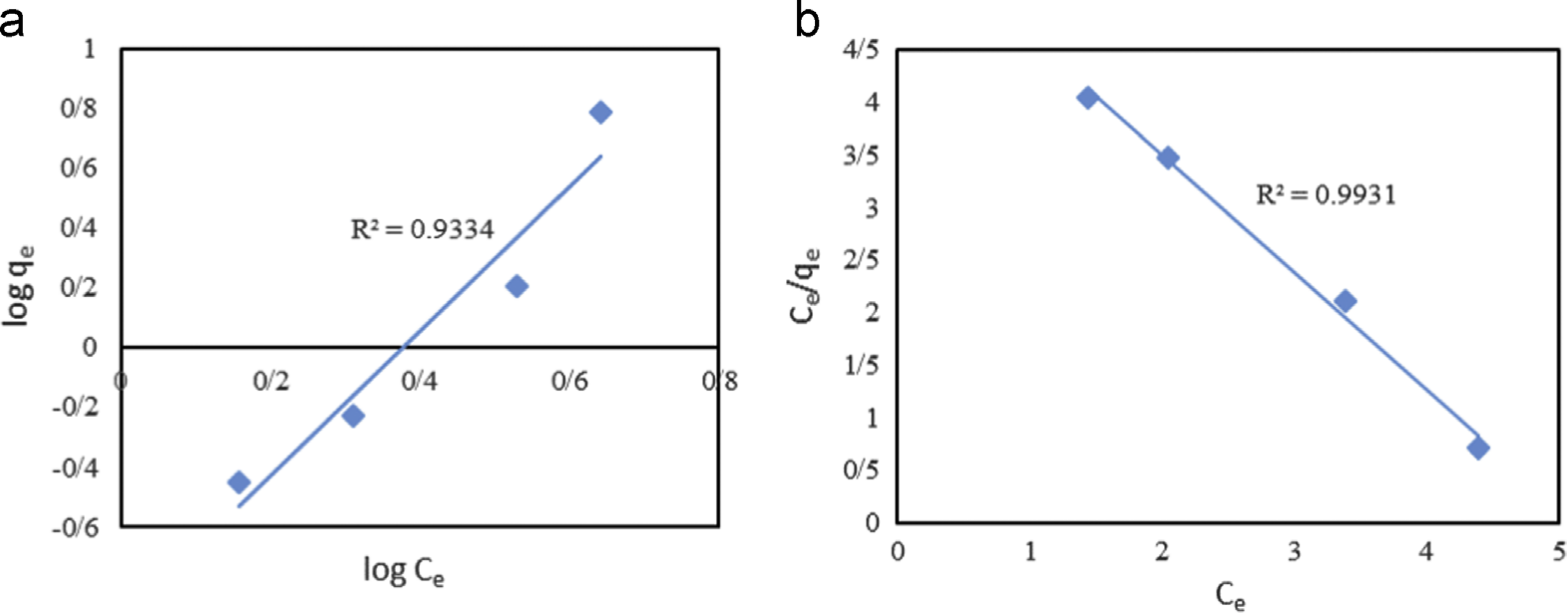
(a) Freundlich, (b) Langmuir isotherms investigation of Fe (II) biosorption by *Sargassum hystrix* algae.

**Fig. 2:**
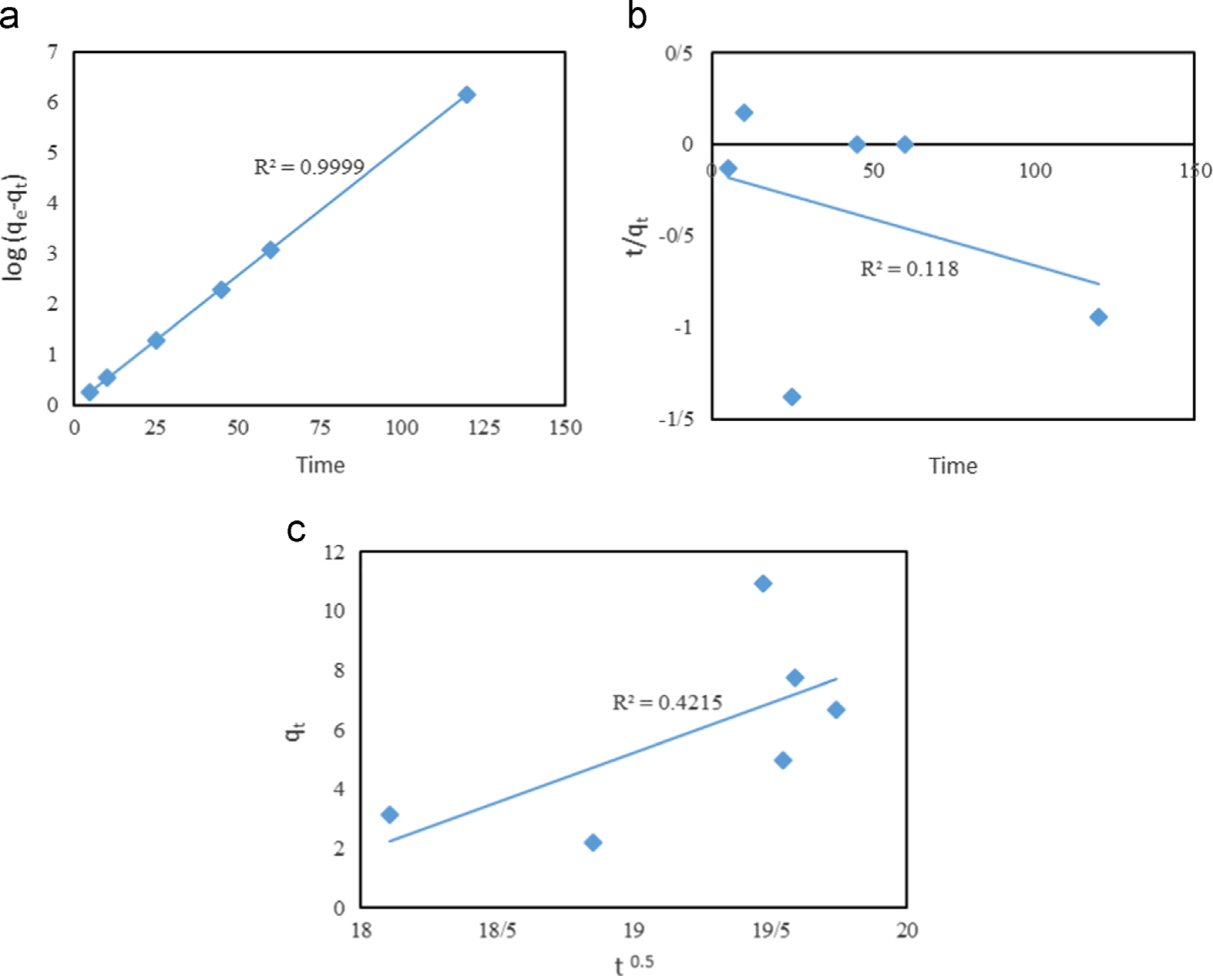
(a) Pseudo-first-order model, (b) Pseudo-second-order model, and (c) intraparticle diffusion kinetic model of Fe (II) biosorption onto *Sargassum hystrix* algae.

**Fig. 3:**
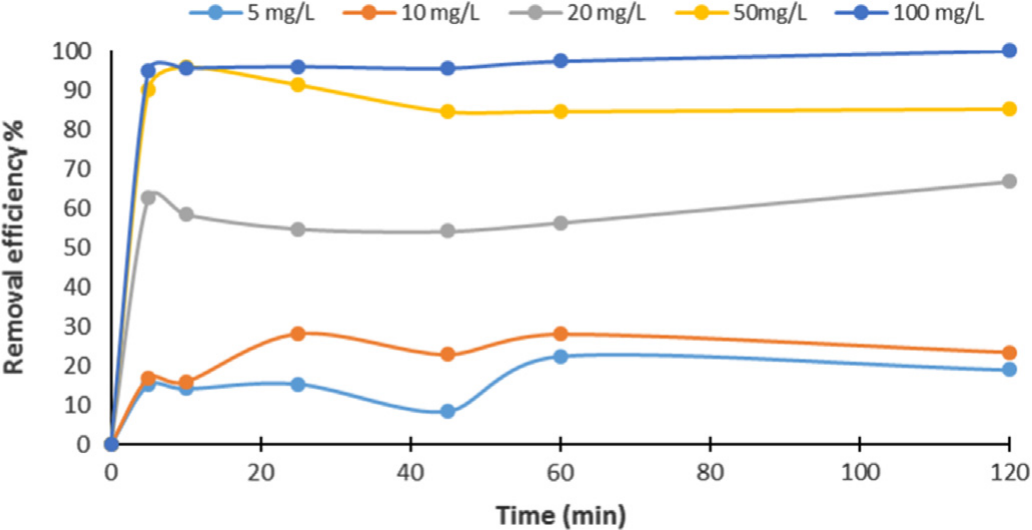
Fe (II) biosorption as a function of initial Fe (II) concentration (pH: 7; biosorbent dose; 10 g/L).

**Fig. 4:**
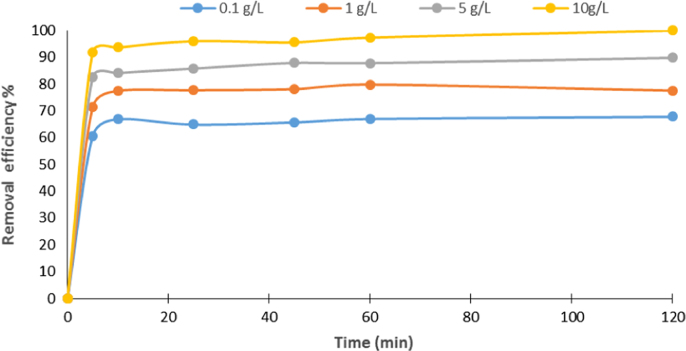
Fe (II) biosorption as a function of biosorbent dose (pH: 7; initial Fe (II) concentration; 100 mg/L).

In this article the data in Table 1 present the isotherm and kinetic equations that used for description of experiments. Calculated values of isotherm and kinetic model parameters were reported in Table 2. Fig. 1, Fig. 2 show data of different isotherm and kinetic models applied in this study. The maximum biosorption efficiency (99.96%) of Fe (II) was obtained at biosorbent dosage of 10 g/L, Fe (II) concentration level of 100 mg/L, and contact time of 120 min. The effects of different parameters on removal efficiency of Fe (II) by biosorbent are shown in Fig. 3, Fig. 4.

## 2 Experimental design, materials and methods

The brown algae *Sargassum hystrix* was used as biosorbent, was obtained along the northern part of the Persian Gulf in Bushehr seaside region. The collected algae was washed 3 times by urban water and 2 times by deionized water to eliminate dirt and contaminants, next dried in oven (at 105 °C for 24 h) and eventually powdered and sieved by using a screen (Mesh no: 25). FeCl_3_.6H_2_O was used for preparing Fe (II) solutions. Fe (II) solutions were prepared at 5, 10, 20, 50, and 100 mg/L concentration from a stock solution (1000 mg/L). At each experiment, 100 ml of Fe (II) solution with special initial concentration of Fe (II) was agitated at 120 rpm. The effects of 6 contact times (5, 10, 25, 45, 60, and 120 min), 5 initial concentrations of Fe (II) (5–100 mg/L) and different dosage of biosorbent (0.1–10 g/L) were studied in the batch runs. Flame atomic absorption spectrometry (FAAS, Varian AA240, Australia) [1], [2], [3], [4] was used to investigate the remaining concentration of Fe (II) in the aqueous solution after each run. Following equation [5], [6] was applied to calculate the removal efficacy during experiments.

$$R=\frac{{(C}_{i}-C_{e})}{C_{i}}\times 100$$

where *R* is the removal efficacy, *C_i_* and *C_e_* are the levels of Fe (II) before and after the experiment in any time (mg/L).

## Figures and Tables

**Table 1:** Isotherm and kinetic equations used in the biosorption of Fe (II) in present study.

**Model**	**Functional form**	**Ploting**
**Langmuir**	$\frac{C_{e}}{q_{e}}=\frac{1}{{\mathrm{bq}}_{\max}}+\frac{1}{q_{\max}}C_{e}$	$\frac{C_{e}}{q_{e}}$ vs *C_e_*
**Freundlich**	Log (*q_e_*)=Log (*K_f_*)+Log *C_e_*	log *q_e_* vs log *C_e_*
**First-order kinetic**	Log (*q_e_*−*q_t_*)=log *q_e_*−$\frac{K_{1,\mathrm{ads}}}{2.303}t$	log (*q_e_*−*q_t_*) vs *t*
**Second-order kinetic**	$\frac{t}{q_{t}}=\frac{1}{{q_{e}^{2}K}_{2,\mathrm{ads}}}+\frac{1}{q_{e}}t$	*t*/*q_t_* vs *t*
**Intraparticle diffusion**	*q_t_*=*k_d_ t*^0.5^+*C*	*q_t_* vs *t*^0.5^

*q_e_* is the mass of Fe (II) biosorbed per unit weight of the biosorbent (mg/g), *q_max_* is the monolayer biosorption capacity, *b* is the Langmuir constant related to the free energy of biosorption equilibrium concentration level of Fe (II) in solution (mg/L) after biosorption and *K_f_* is the Freundlich capacity factor and a measure of biosorption capacity, 1/*n* is the equilibrium concentration level of Fe (II) in solution (mg/L) after biosorption, *q_t_* (mg/g) is the amount of biosorbed Fe (II) on algae at equilibrium and time *t* (min), *C* is the intercept and *k*_1_ (1/min), *k*_2_ (g/mg min) and *k_d_* (mg/g min^0.5^) are the rate constants of pseudo-first order, pseudo second order kinetic and intraparticle diffusion model.

**Table 2:** Isotherm and kinetic parameters for Fe (II) biosorption onto *Sargassum hystrix* algae.

**Isotherms and kinetics models**	**Parameter**	**Value**
**Langmuir**	*b* (L/mg)	0.194
	*R_L_*	0.507
	*q_max_* (mg/g)	0.894
	*R*^2^	0.993

**Freundlich**	*K_f_* (mg/g)	0.121
	1/*n*	2.425
	*R*^2^	0.933

**First-order kinetic**	*q*_*e* cal_(mg/g)	10.18
	*q*_*e* exp_(mg/g)	19.59
	*K*_1_ (1/min)	0.117
	*R*^2^	0.999

**Second-order kinetic**	*q*_*e* cal_ (mg/g)	196.07
	*q*_*e* exp_(mg/g)	10.18
	*K*_2_ (g/mg min)	0.032
	*R*^2^	0.118

**Intraparticle diffusion**	*K_d_*	3.323
	*C*	57.902
	*R*^2^	42.15
